# Myoblast cytonemes mediate Wg signaling from the wing imaginal disc and Delta-Notch signaling to the air sac primordium

**DOI:** 10.7554/eLife.06114

**Published:** 2015-05-07

**Authors:** Hai Huang, Thomas B Kornberg

**Affiliations:** 1 Cardiovascular Research Institute, University of California, San Francisco, San Francisco, United States; National Centre for Biological Sciences, Tata Institute for Fundamental Research, India

**Keywords:** cytoneme, Notch, Delta, Wingless, frizzled, diaphanous, *D. melanogaster*

## Abstract

The flight muscles, dorsal air sacs, wing blades, and thoracic cuticle of the *Drosophila* adult function in concert, and their progenitor cells develop together in the wing imaginal disc. The wing disc orchestrates dorsal air sac development by producing decapentaplegic and fibroblast growth factor that travel via specific cytonemes in order to signal to the air sac primordium (ASP). Here, we report that cytonemes also link flight muscle progenitors (myoblasts) to disc cells and to the ASP, enabling myoblasts to relay signaling between the disc and the ASP. Frizzled (Fz)-containing myoblast cytonemes take up Wingless (Wg) from the disc, and Delta (Dl)-containing myoblast cytonemes contribute to Notch activation in the ASP. Wg signaling negatively regulates Dl expression in the myoblasts. These results reveal an essential role for cytonemes in Wg and Notch signaling and for a signal relay system in the myoblasts.

**DOI:** http://dx.doi.org/10.7554/eLife.06114.001

## eLife digest

Fruit fly larvae undergo a remarkable physical transformation to become an adult fly. During this transformation, the tissues in the larvae change into the structures found in the adult. For example, the adult wings, flight muscles, and other structures needed for coordinated flight form from a pair of disc-like tissues called the wing imaginal discs.

For these structures to develop correctly, the cells in the wing imaginal discs need to receive coordinated instructions about what types of cells they need to become. Within the wing discs, finger-like projections called cytonemes link specific cells together to allow signal molecules to move between the cells; this controls the development of the wing disc itself as well as structures called dorsal air sacs, which supply oxygen to the flight muscles in the adult fly. However, it is not known if cytonemes allow the exchange of signal molecules between cells involved in the formation of other structures needed for flight.

Here, Huang and Kornberg investigated the role of cytonemes in the development of the flight muscles in fruit flies. The experiments reveal that cells called myoblasts—which will later become the flight muscle cells—form two sets of cytonemes with other cells. One set connects the myoblasts to cells in the developing air sac, which allows a signal protein called Delta to signal from the myoblasts into the air sac cells. The other set of cytonemes connects the myoblasts to wing disc cells. This enables another signal molecule called Wingless, which is produced in wing disc cells, to move into the myoblasts and block the production of Delta.

Huang and Kornberg's findings reveal a new role for cytonemes in coordinating the development of the flight muscles and the dorsal air sacs. A future challenge will be to understand how individual cytonemes are able to connect to specific cells.

**DOI:** http://dx.doi.org/10.7554/eLife.06114.002

## Introduction

Flight muscles of the *Drosophila* adult drive the coordinated movements of the wings and thoracic cuticle to power flight, and many thin tubes (tracheoles) that emanate from the thoracic dorsal air sacs penetrate the muscles to oxygenate them. Thus, the functions of the muscles, wings, thoracic cuticle, and trachea are linked, and the physical associations are intimate. The progenitor cells that produce these tissues develop together in the wing imaginal disc. Previous studies from this lab showed that the air sac primordium (ASP), which is the progenitor of the dorsal air sacs, depends on Branchless/FGF (FGF) and Dpp signaling proteins that the wing disc produces (Sato and Kornberg, 2002; Roy et al., 2014). Here, we describe two other signaling systems that coordinate the progenitors of the flight muscles with the wing disc and trachea.

The wing disc can be described as a flattened sac that juxtaposes the apical surfaces of two connected epithelial sheets across a narrow lumen. One of the sheets, called the columnar epithelium because its cells are highly elongated along their apical/basal axis, generates the wing blade and most of the notum, the dorsal cuticle of the thorax. The wing disc is encapsulated by a basement membrane, but a branch of the tracheal system (the transverse connective) penetrates the basement membrane at several sites in the dorsal region of the disc (Guha et al., 2009). Transverse connective that is within the basement membrane lies adjacent to the basal surface of the columnar epithelium, and during the third instar (L3), this segment of the transverse connective sprouts a tubular outgrowth—the ASP—in response to FGF expressed by a group of nearby columnar epithelial cells (Sato and Kornberg, 2002). Myoblasts that are the progenitors of the flight muscles are also at the basal surface of the columnar epithelium, underneath the basement membrane, and in the vicinity of the tracheal branches. They proliferate during L3 to extend over most of the dorsal part of the disc where the cells that will produce the notum cuticle grow (Sudarsan et al., 2001; Gunage et al., 2014).

Signaling proteins that contribute to the growth and diversification of the cells of the wing disc have been extensively characterized. Three that are relevant to the ASP and myoblasts are Notch, Dpp, and Wg (Couso et al., 1995; Ng et al., 1996; Brennan et al., 1999; Steneberg et al., 1999; Sudarsan et al., 2001; Baena-Lopez et al., 2003; Giraldez and Cohen, 2003; Marois et al., 2006; Herranz et al., 2008; Gunage et al., 2014). Notch signaling has essential roles at both the dorsal/ventral and anterior/posterior compartment borders of the disc, and although it has been shown to specify fusion cell fate and branch identity during formation of tracheal system in the embryo, a role in larval trachea has not been reported. Studies in several other contexts indicate that Notch signaling may be mediated by cytonemes that make direct contacts between signaling cells (Renaud and Simpson, 2001; Cohen et al., 2010).

Dpp-expressing cells line the anterior side of the anterior/posterior compartment border at all stages of L3 discs, and Dpp that is produced near the ASP activates Dpp signal transduction in the ASP that is necessary for its morphogenesis. ASP cells express the Dpp receptor but do not express Dpp. The mechanism by which Dpp signals from disc cells to the ASP involves exchange of Dpp between producing and receiving cells at synapses that form where cytonemes link ASP cells to Dpp-producing disc cells (Roy et al., 2014). ASP cytonemes that contain the Dpp receptor have been observed extending as far as 40 μm, crossing over approximately 15–20 disc cells to reach sources of Dpp. These cytonemes transport Dpp from producing cells to the ASP, and signal transduction is dependent on the contacts they make with the disc cells. Comparably long ASP cytonemes containing the FGF receptor have been observed reaching FGF-expressing disc cells, and in the wing disc, Hh dispersion is effected by a similar mechanism (Callejo et al., 2011; Bischoff et al., 2013). In these contexts, the evidence that Dpp, FGF, and Hh paracrine signaling are mediated by cytonemes is strong.

Expression patterns of Wg change throughout the L3. In the wing blade primordium, Wg is expressed broadly in early L3 discs, but in late L3 discs, it is expressed in well-delineated bands both at the dorsal/ventral compartment border and around the periphery (Phillips and Whittle, 1993; Couso et al., 1995; Strigini and Cohen, 2000; Alexandre et al., 2014). Wg-expressing cells at the dorsal/ventral border may function as a signaling center for the growth and diversification of the wing cells. Wg is also expressed in the notum primordium where it has a role in myoblast proliferation and diversification (Sudarsan et al., 2001; Gunage et al., 2014).

The mechanism by which Wg signals from the disc cells to myoblasts has not been studied. In other contexts, it has been assumed that Wg is secreted and released by producing cells, and that it reaches target cells by passive diffusion, and models that describe its movement posit that its path may be restricted by structures it encounters, but that its hypothetical journey is similar to a ‘drunken sailor’ (Muller et al., 2013). In wing discs, a mutant form of Wg that is tethered to the plasma membrane by a heterologous transmembrane domain is active (Alexandre et al., 2014), but it is not known how signaling by this tethered form is related to the normal processes that present wild type Wg to receiving cells. There is no experimental evidence that shows directly that Wg disperses by passive diffusion or that it signals in vivo as a soluble, free protein. The study reported here investigated signaling between disc cells, myoblasts, and the ASP. The Wg-Fz and Notch-Dl signaling systems were identified as important to ASP development, and two types of myoblast cytonemes were characterized that mediate exchange and transport of Wg and signaling by Dl.

## Results

### Trachea and myoblasts in the third instar wing disc

**Figure 1.**
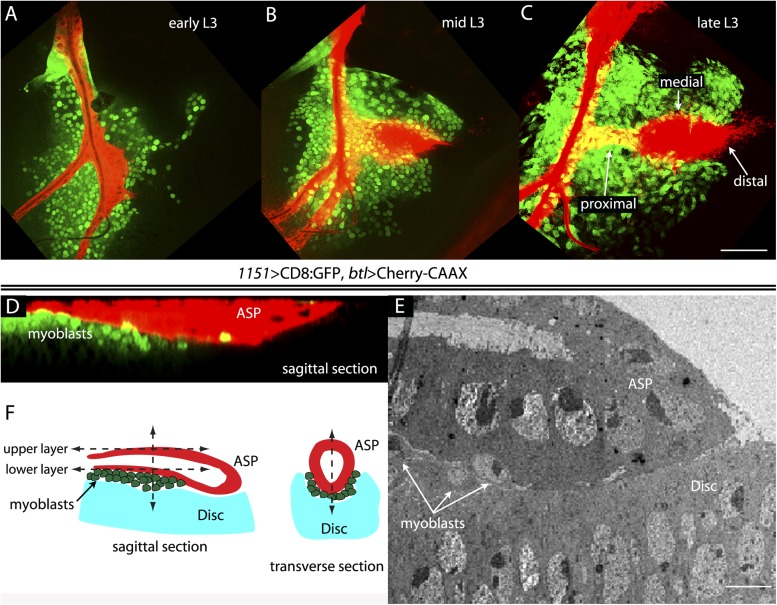
The close proximity of wing disc myoblasts and the ASP. (**A**–**C**) Confocal images show the trachea and myoblasts at early (**A**), mid (**B**), and late (**C**) L3 stages. (Genotype: *1151-Gal4/+;btl-LHG/+;UAS-CD8GFP/lexO-mCherry-CAAX*). Scale bar: 50 μm. (**D**) Sagittal cross-section shows myoblasts adjacent to the proximal portion of the ASP. Genotype as in (**A**–**C**). Scale bar: 50 μm. (**E**) Electron microscopic image shows a sagittal view of the wing disc columnar epithelium, associated myoblasts (white arrows), and the ASP. Scale bar: 5 μm. (**F**) Cartoon showing a cross-section of the ASP (red) and wing disc epithelium (blue). In the left drawing, dotted lines represent the approximate positions of upper and lower layers of ASP and the vertical dashed line corresponds to the location of the transverse optical section on the right and in Figure 2B. Vertical dashed line in transverse section corresponds to plane imaged in Figure 2B′. **DOI:** http://dx.doi.org/10.7554/eLife.06114.003

To investigate the relative proximity of the myoblasts and trachea as they grow and develop during L3, we simultaneously expressed membrane-tethered mCherry in the trachea (with *btl-LHG lexO-mCherry:CAAX*, a tracheal-specific driver [Shiga et al., 1996]) and membrane-tethered GFP in myoblasts (with *1151-Gal4 UAS-CD8:GFP*; a myoblast-specific driver [Roy and VijayRaghavan, 1997, 1998]), and monitored both fluorescent proteins in disc preparations. During L3, the myoblasts increase in number from approximately 250 to 2500 (Gunage et al., 2014), and the ASP buds from the transverse connective to grow posteriorly across the anterior compartment. Images of the mid-dorsal region of early, mid, and late L3 discs revealed the expansion of the myoblasts and growth of the ASP as well as the close proximity of the trachea and myoblasts (Figure 1A–C). These figures also show that the distal ASP extended beyond the myoblast domain; cytonemes that emanate from the distal tip and that take up FGF from wing disc (Sato and Kornberg, 2002; Roy et al., 2011, 2014) were also visible (arrows). The close proximity of the ASP and myoblasts is also apparent in a sagittal view (Figure 1D) and in an electron micrograph image (Figure 1E). The drawing in Figure 1F depicts the ASP, myoblasts, and disc cells at the late L3.

**Figure 2.**
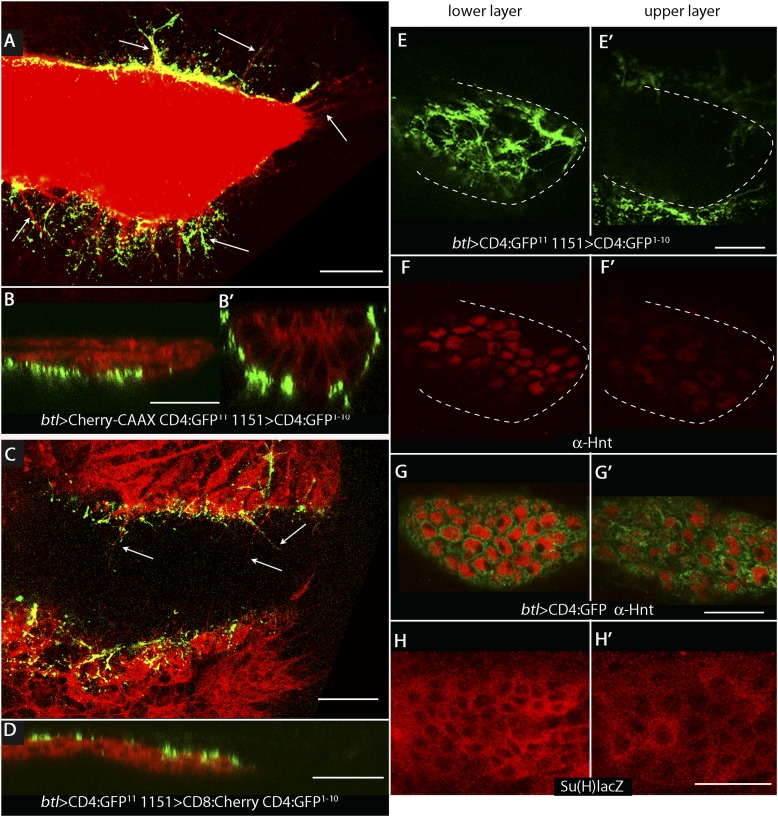
Wing disc myoblasts contact the lower layer of the ASP. (**A**) Medial optical section, (**B**) sagittal plane and (**B′**) transverse plane of the ASP (red, Cherry fluorescence) with green fluorescence indicating reconstituted GFP at regions of contact between myoblasts that expressed GFP^1–10^ and the ASP that expressed GFP^11^. Cytonemes (arrows in [**A**]) extend from the ASP; green fluorescence marks cytonemes that contact myoblasts. (Genotype: *1151-Gal4/+;btl-LHG, lexO-mCherry-CAAX/lexO-CD4-GFP*^*11*^*;UAS-CD4-GFP*^*1–10*^*/+*). (**C**) Medial optical section and (**D**) sagittal plane of a preparation similar to (**A** and **B**), but with the myoblasts marked with Cherry fluorescence. Myoblast cytonemes extend to the ASP; green fluorescence marks cytonemes that contact the ASP. (Genotype: *1151-Gal4/+;UAS-CD8-mCherry/btl-LHG;UAS-CD4-GFP*^*1–10*^ *lexO-CD4-GFP*^*11*^*/+*). (**E**–**H′**) Upper (left panels) and lower (right panels) optical sections (see Figure 1F) show GFP reconstitution across synaptic partners (GRASP) fluorescence (**E** and **E′**), staining with α-Hnt antibody (**F**–**G′**), and staining with α-ß-galactosidase antibody (**H** and **H′**). GRASP fluorescence, Hnt levels and Su(H)lacZ levels were higher in the lower than in the upper layer of the ASP. Scale bars: 25 μm. **DOI:** http://dx.doi.org/10.7554/eLife.06114.004

To investigate whether the ASP, myoblasts, and disc cells contact each other directly, we applied the GFP reconstitution across synaptic partners (GRASP) technique (Feinberg et al., 2008). This method monitors the interaction of two non-fluorescent fragments of GFP (GFP^1–10^ and GFP^11^) that are expressed separately as external, membrane-tethered proteins on different cells; reconstitution of GFP by fragments present on cells that juxtapose at <100 nm generates green fluorescence. We co-expressed mCherry-CAAX (to label ASP membranes) and CD4:GFP^11^ in the ASP, and expressed CD4:GFP^1–10^ in myoblasts. As shown in panels A, B, and B′ in Figure 2, GFP fluorescence was present at the periphery of the ASP. Most of the GRASP fluorescence was at proximal and medial regions of the ASP, and little was near the distal tip, consistent with the observed distribution of myoblasts (Figure 1D–F). ASP cytonemes marked with mCherry were visible that extended as much as 25 μm (approximately 5 myoblast cell diameters) from the ASP periphery. Whereas most of the GFP fluorescence on the dorsal side (downward orientation in Figure 2A) was associated with these cytonemes, most of the ventral fluorescence was close to the ventral edge of the ASP.

A ‘reciprocal’ experiment imaged ASP-myoblast GRASP in the context of myoblasts that were marked with mCherry, and this genotype generated similar patterns of GFP fluorescence. Both GRASP contexts illuminated contacts between the ASP and myoblasts that can be seen in frontal optical sections (Figure 2A,C) and in sagittal projections of optical sections at the middle of the ASP (Figure 2B,D; see Figure 1F). Transverse projections of optical sections at the middle of the ASP (Figure 2B′; see Figure 1F) show that the ASP lays in a depression of the disc's basal surface and that ASP cells at the ‘lowest level’ (‘6 o'clock’ in transverse sections as in Figures 1F, 2B′) contacted myoblasts. The transverse projection (Figure 2B′) also reveals contacts between myoblasts and cells at lateral positions around the ASP circumference. GRASP fluorescence in frontal sections (Figure 2E,E′) at the lower and upper layers of the ASP (as defined in Figure 1F) confirms this pattern of contacts. In addition, mCherry fluorescence marked myoblast cytonemes, revealing that cytonemes that link myoblasts to the ASP extended from both types of cells (Figure 2A,C).

### Notch signaling between myoblasts and the ASP

Previous studies showed that Dpp signal transduction is elevated in cells in the ‘lower layer’ of the ASP and is reduced in the ‘upper layer’, a pattern that correlates with GRASP-marked contacts between ASP cytonemes and Dpp-expressing disc cells (Roy et al., 2014). In order to identify the signaling system that correlates with the contacts between myoblasts and cells in the ‘lower layer’ of the ASP, we examined the expression patterns of several candidate genes. We did not detect expression of Senseless, Vestigial, Cut, or Distal-less in the ASP, and although we detected expression of Escargot, Trachealess, and Pointed, there was no apparent difference in expression levels between the ‘upper’ and ‘lower layers’. In contrast, we found that both the intensity of staining with α-Hindsight (Hnt) antibody and the expression of Su(H)GBE-lacZ (Su(H)lacZ) were significantly greater in the ‘lower layer’ (Figure 2F–H′). Hnt and Su(H)lacZ are targets of Notch signaling (Furriols and Bray, 2001; Bray, 2006; Sun and Deng, 2007), suggesting that Notch signaling may be activated in the ASP and may be associated with contacts between myoblasts and the ASP.

**Figure 3.**
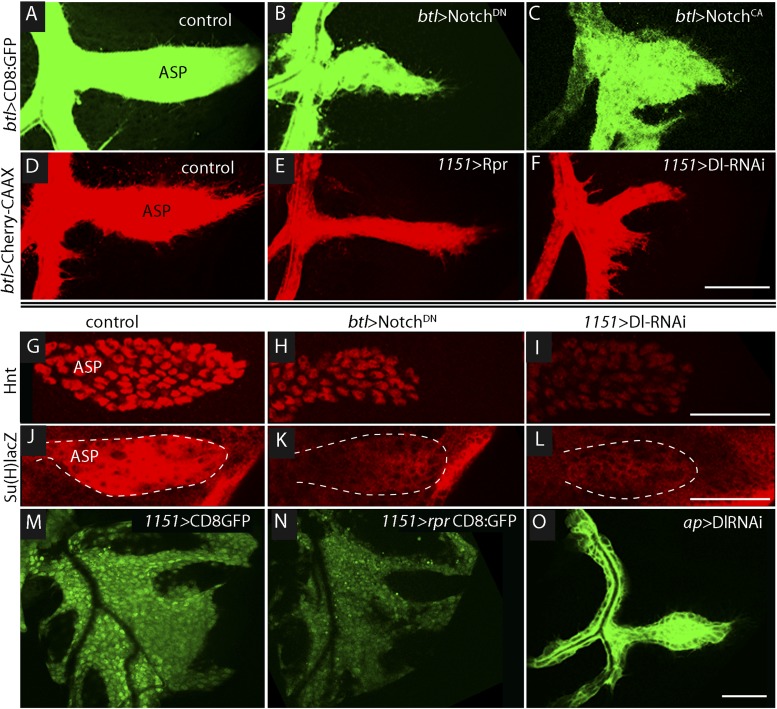
Notch signaling in the ASP depends upon myoblast-produced Delta. (**A**–**F**) Confocal images show that compared to controls (**A** and **D**), the ASP morphology was abnormal under conditions that either knockdown (**B**) or stimulate (**C**) Notch signaling, or that perturb myoblasts by expression of Rpr (**E**) or *DlRNAi* (**F**). Animals were reared at 18°C for 4 days (to L2 stage) and shifted to 29°C for >48 hr (to late L3 stage). (Genotypes: (**A**) *btl-Gal4 UAS-CD8:GFP/+;tub-Gal80*^*ts*^*/+*; (**B**) *btl-Gal4, UAS-CD8:GFP/UAS-Notch*^*DN*^*;tub-Gal80*^*ts*^*/+*; (**C**) *btl-Gal4, UAS-CD8:GFP/UAS-Notch*^*CA*^*;tub-Gal80*^*ts*^*/+*; (**D**) *1151-Gal4/+;btl-LHG lexO-Cherry:CAAX/+*; (**E**) *1151-Gal4/+;btl-LHG lexO-Cherry:CAAX/UAS-rpr*; and (**F**) *1151-Gal4/+;btl-LHG lexO-Cherry:CAAX/+;UAS-DlRNAi/+*). (**G**–**I**) Compared to controls (**G** and **J**), levels of Hnt and Su(H)lacZ expression were lower under conditions that reduce Notch activity in ASP cells (**H** and **K**) or reduce Dl expression in myoblasts (**I** and **L**). Staining was with α-Hnt (**G**–**I**) and α-ß-galactosidase (**J**–**L**) antibodies and Alexa Fluor 555 secondary antibodies. ASPs are outlined by dotted lines. (**M** and **N**) The number of myoblasts (marked with CD8:GFP) and intensity of GFP fluorescence were reduced by expression of Rpr (**N**) relative to control (**M**). (**O**) Expression of DlRNAi in the wing disc had no apparent effect on ASP development. (Genotypes: (**G**) *1151-Gal4/+*; (**H**) *btl-Gal4/UAS-Notch*^*DN*^*;tub-Gal80*^*ts*^*/+*; (**I**) *1151-Gal4/+;UAS-DlRNAi/+*; (**J**) *Su(H)lacZ/+;btl-Gal4/+*, (**K**) *Su(H)lacZ/+;UAS-Notch*^*DN*^*/+;btl-Gal4/tub-Gal80*^*ts*^; (**L**) *Su(H)lacZ/1151-Gal4;UAS-DlRNAi/+*; (**M**) *1151-Gal4/+;UAS-CD8:GFP/+*; (**N**) *1151-Gal4/+;UAS-rpr/+;UAS-CD8:GFP/+*; (**O**) *ap-Gal4/+;btl-LHG lexO-CD8:GFP/DlRNAi*). Scale bars: 50 μm. **DOI:** http://dx.doi.org/10.7554/eLife.06114.005

To determine if Notch signaling has a role in tracheal development, we altered Notch signaling in several ways, including expressing dominant-negative Notch (Notch^DN^), constitutive active Notch (Notch^CA^), and Dl-RNAi. In comparison with controls (Figure 3A,D), the ASP was reduced in size by tracheal expression of Notch^DN^ (Figure 3B) and was misshapen and expanded following tracheal expression of Notch^CA^ (Figure 3C). These effects suggest that Notch activity is necessary for ASP development. To determine if Notch signaling in the ASP involves the disc-associated myoblasts, we over-expressed the pro-apoptotic cell death gene *reaper* (*rpr*) in myoblasts to reduce their number (Figure 3M,N). ASP growth was impaired by the *rpr* over-expression (Figure 3E). We then investigated a role for Delta (Dl) because it is a Notch ligand that is highly expressed in myoblasts (Gildor et al., 2012). To test whether the myoblasts produce Delta that signals to activate Notch in the trachea, Delta was depleted by expression of Dl-RNAi in myoblasts; abnormally small and misshapen ASPs were observed (Figure 3F). In contrast, expression of Dl-RNAi in the disc had no apparent effect on the ASP (Figure 3O).

Notch signaling in the ASP was monitored by assaying the Notch reporters Hnt and Su(H)lacZ. Compared to control ASPs, levels of Hnt and Su(H)lacZ in the lower layer of the ASP (where Notch is activated [Figure 2F–H′]) were reduced by expression of Notch^DN^ in trachea and by expression of Dl-RNAi in the myoblasts (Figure 3G–L). These data suggest that Dl expressed in myoblasts activates Notch signaling in the ASP, and that this signaling system is necessary for ASP development.

### Myoblast cytonemes present Delta to the ASP

**Figure 4.**
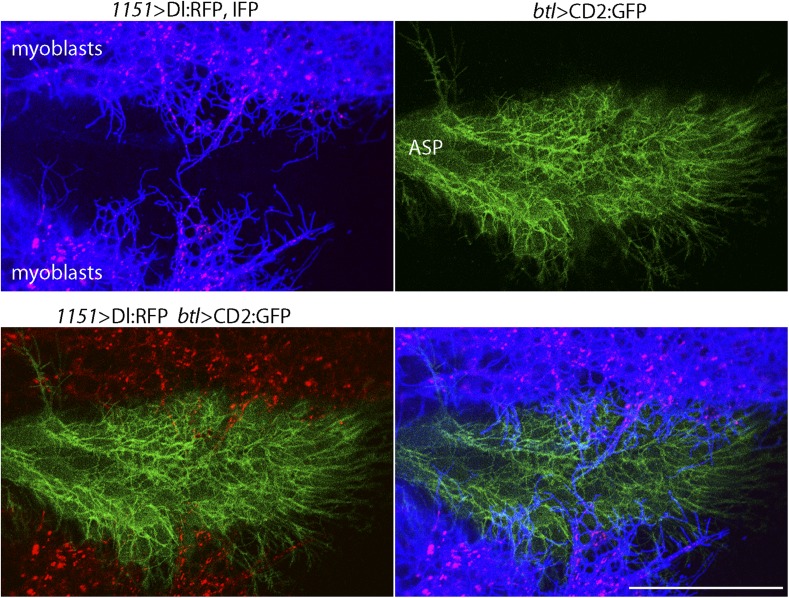
Delta localizes to myoblast cytonemes. Confocal image of a late L3 ASP marked with CD2:GFP (*btl-LHG lexO-CD2:GFP*) and myoblasts marked with CD4:IFP (*1151-Gal4 UAS-CD4:IFP2.0-HO1*) and that express Dl:RFP (*1151-Gal4 UAS-Dl:RFP*). Dl:RFP puncta in IFP-marked myoblast cytonemes and CD2:GFP-containing ASP cytonemes are visible extending across the basal surface of the lower layer of the ASP. Scale bar: 50 μm. **DOI:** http://dx.doi.org/10.7554/eLife.06114.006

**Figure 5.**
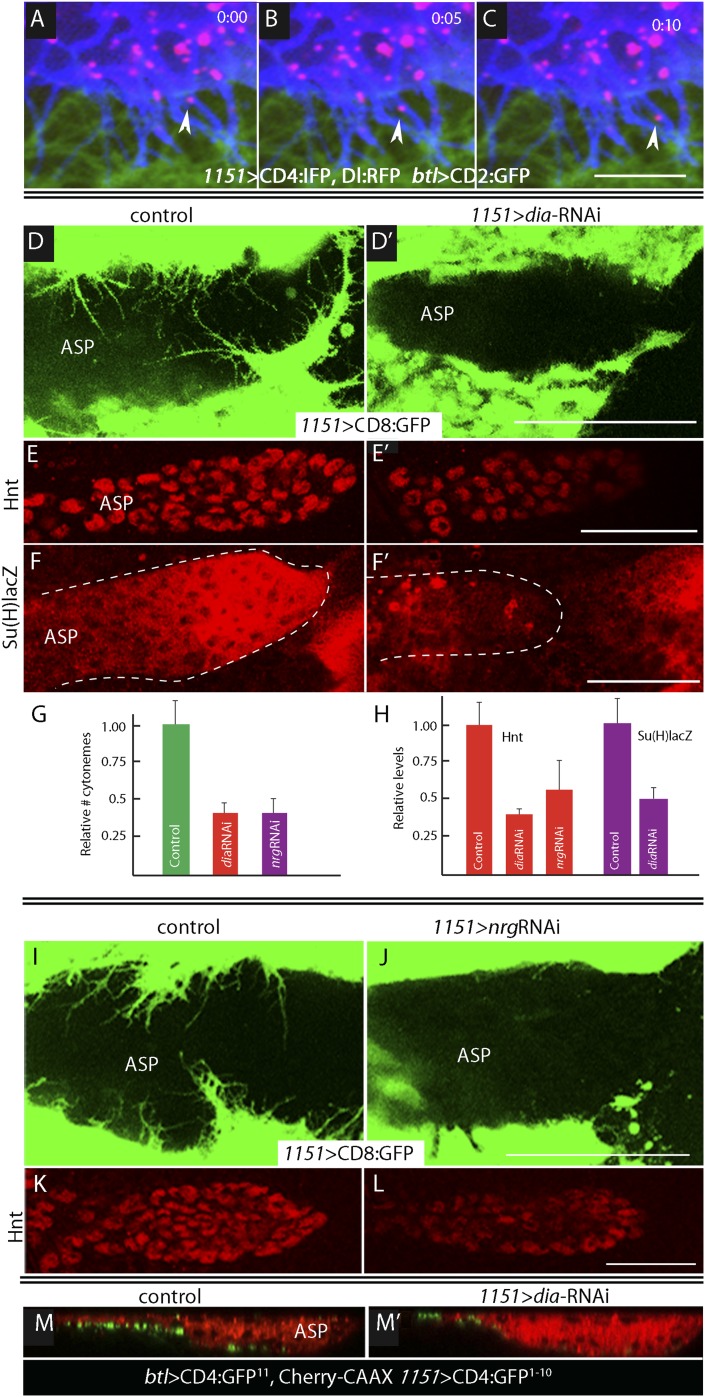
Delta is motile in myoblast cytonemes and activates Notch signaling in the ASP. (**A**–**C**) Confocal images of a late L3 ASP marked with CD2:GFP (*btl-LHG lexO-CD2:GFP*) and myoblasts marked with CD4:IFP (*1151-Gal4 UAS-CD4:IFP2.0-HO1*) and that express Dl:RFP (*1151-Gal4 UAS-Dl:RFP*). Dl:RFP puncta are visible in myoblasts and myoblast cytonemes. Images taken 5 s apart captured the motion of Dl-containing puncta (arrowheads in **A**, **B**, **C**). Scale bar: 10 μm. (**D**–**D′**) Projection images in control (*1151-Gal4/+;UAS-CD8:GFP/+*) and *diaRNAi* (*1151-Gal4/+;UAS-CD8:GFP/UAS-diaRNAi*) flies show that myoblasts with reduced *dia*RNA had fewer cytonemes. (**E**–**F′**) ASPs with the same genotypes as (**D** and **D**′) stained with α-Hnt (**E** and **E′**) and α-ß-galactosidase antibodies (**F** and **F′**) and with Alexa Fluor 555 secondary antibodies show that both Hnt and Su(H)lacZ were reduced under conditions of *diaRNAi*. (**G** and **H**) Bar graphs showing the dependence on dia and nrg function of myoblast cytoneme numbers per unit length of the imaged circumference of the ASP (**G**), and (**I**) Hnt and Su(H)lacZ levels (measured as fluorescence intensity in arbitrary units). p values, (**G**) 7.17E-12 and 8.475E-08 for *diaRNAi* and *nrgRNAi*, respectively; (**H**) 3.48E-05 and 1.79E-03 for *diaRNAi* and *nrgRNAi* levels of Hnt, respectively, and 5.9E-04 for *diaRNAi* levels of Su(H)lacZ; error bars: standard deviation. (**I**–**L**) Myoblast cytonemes (marked with CD8:GFP) that extend across the ASP were reduced in the presence of nrgRNAi (**I** and **J**); and in the ASP, the level of α-Hnt staining (a readout of Delta-Notch signaling) was also reduced (**K** and **L**). Genotypes: control (*1151-Gal4/+;UAS-CD8:GFP/+*) and *nrgRNAi* (*1151-Gal4/+;UAS-nrgRNAi/+;UAS-CD8:GFP/+*). (**M** and **M′**) Sagittal views of ASPs (red, Cherry fluorescence) show that contacts between myoblasts and the ASP marked by GRASP fluorescence were reduced in *dia-*RNAi (**M′**; *1151-Gal4/+;UAS-CD4:GFP*^*1–10*^ *lexO-CD4:GFP*^*11*^*/btl-LHG, lexO-mCherry-CAAX;UAS-diaRNAi/+*) flies compared to controls (**M**; *1151-Gal4/+;UAS-CD4:GFP*^*1–10*^ *lexO-CD4:GFP*^*11*^*/btl-LHG lexO-Cherry:CAAX*). Scale bars: 50 μm. **DOI:** http://dx.doi.org/10.7554/eLife.06114.007

We investigated whether the myoblast cytonemes that contact the ASP (Figure 2C) may mediate signaling by myoblast-produced Dl. To track myoblast-produced Dl, we expressed Dl:RFP together with a fluorescent membrane protein marker (CD4:IFP2.0-HO1) in the myoblasts (Yu et al., 2014), and in the same animals expressed membrane-tethered GFP in the ASP. In the plane of focus captured in the images in Figure 4, cytonemes with GFP fluorescence are visible at the surface of the ASP, and cytonemes with IFP fluorescence are visible that emanated from the myoblasts. Many Dl:RFP-containing puncta are visible in the myoblasts, and many of these fluorescent puncta colocalized with and moved along IFP-marked cytonemes. The motile puncta indicated in Figure 5A–C and visible in Video 1 moved at 0.33 μm/s, a speed that is consistent with rates of myosin motors.

**Video 1.** Motile Delta-RFP in myoblast cytonemes. Dl:RFP expressed in myoblasts was detected in fluorescent puncta that moved along dynamic myoblast cytonemes (labeled by CD4:IFP2.0–HO1); the ASP was labeled by CD2:GFP. (Genotype: *1151-Gal4/+;btl-LHG lexO-CD2:GFP/+; UAS-Dl:RFP UAS-CD4:IFP2.0-HO1/+*.) **DOI:** http://dx.doi.org/10.7554/eLife.06114.008

To investigate whether the IFP-marked, Dl-containing cytonemes function in Notch signaling, we genetically ablated myoblast cytonemes by inactivating *diaphanous* (*dia*) and *neuroglian* (*nrg*). The Dia protein is a formin whose activated form localizes to tips of filopodia (Homem and Peifer, 2008; Rousso et al., 2013) and cytonemes (Roy et al., 2014), and Nrg is an L1-type cell adhesion protein that has been implicated in the development and stability of neuronal synapses (Enneking et al., 2013). Loss-of-function phenotypes are dependent on context and degree of knockdown and negative results are uninformative, but it has been reported that Dia and Nrg are necessary for cytoneme-mediated Dpp signaling in the ASP (Roy et al., 2014). We examined the effects of *dia*RNAi and *nrg*RNAi expression on myoblast cytonemes and on Notch signaling in the ASP and found that both the relative number of myoblast cytonemes and Notch signal transduction in the ASP were reduced (Figure 5D–L). Notch signaling was monitored by expression of Hnt (for *dia*RNAi and *nrg*RNAi) and Su(H)lacZ (for *dia*RNAi). Expression of *dia*RNAi reduced contacts between myoblasts and the ASP (as indicated by reduced GRASP fluorescence; Figure 5M,M′). These results suggest that the Dl-containing myoblast cytonemes present Delta to the ASP and make contacts that activate Notch signaling in the ASP.

### Endocytosis is required for Delta function in myoblasts

**Figure 6.**
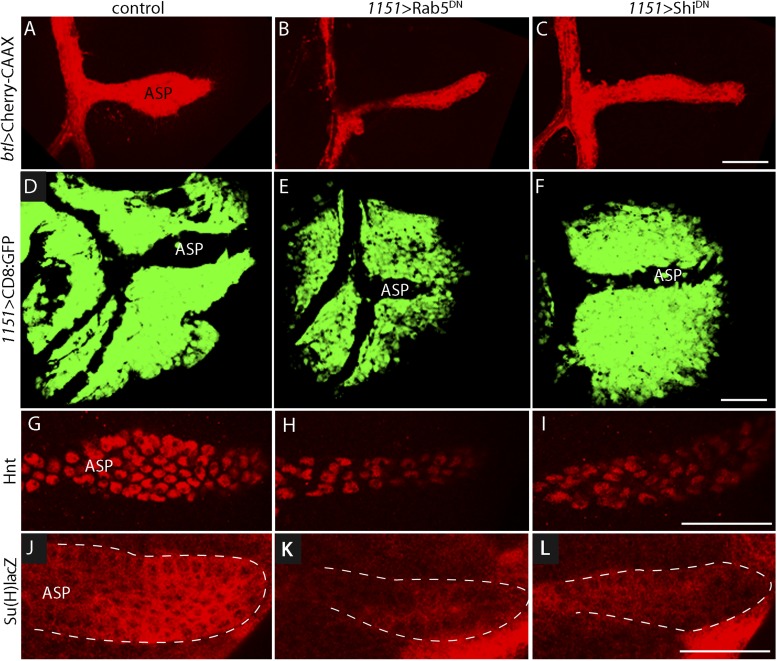
Myoblast functions necessary for Notch signaling in the ASP. Compared to controls (**A**, **D**, **G**, **J**), reduction of Rab5 function (**B**, **E**, **H**, **K**) or of Dynamin function (**C**, **F**, **I**, **L**) perturbed ASP development (**B**, **C**, **E**, **F**) and reduced Hnt (**H** and **I**) and Su(H)lacZ (**K** and **L**) expression. (**G**–**L**) Staining was with α-Hnt (**G**–**I**) and α-ß-galactosidase antibodies (**J**–**L**) and Alexa Fluor 555 secondary antibodies. (Genotypes: (**A**) *1151-Gal4/+;btl-LHG, lexO-mCherry-CAAX/+;* (**B**) *1151-Gal4/+;btl-LHG, lexO-Cherry:CAAX/+;UAS-Rab5*^*DN*^*/+*; (**C**) *1151-Gal4/+;btl-LHG, lexO-Cherry:CAAX/+;UAS-Shi*^*DN*^*/+*; (**D**) *1151-Gal4/+;UAS-CD8:GFP/+*; (**E**) *1151-Gal4/+;UAS-CD8:GFP/UAS-Rab5*^*DN*^; (**F**) *1151-Gal4/+;UAS-CD8:GFP/UAS-Shi5*^*DN*^; (**G**) *1151-Gal4/+*; (**H**) *1151-Gal4/+;UAS-Rab5*^*DN*^*/+*; (**I**) *1151-Gal4/+;UAS-Shi*^*DN*^*/+;* (**J**) *Su(H)lacZ/1151-Gal4*; (**K**) *Su(H)lacZ/1151-Gal4;UAS-Rab5*^*DN*^*/+;* (**L**) *Su(H)lacZ/1151-Gal4;UAS-Shi*^*DN*^*/+*). Scale bars: 50 μm. **DOI:** http://dx.doi.org/10.7554/eLife.06114.009

Notch signal transduction involves internalization of activating ligands by ligand-producing cells (Seugnet et al., 1997; Fischer et al., 2006; Windler and Bilder, 2010). To test whether endocytosis by myoblasts is required for Notch signaling in the ASP, we disrupted endocytosis in myoblasts by expressing dominant-negative forms of the Rab5 GTPase (Vaccari et al., 2008; Windler and Bilder, 2010) and the dynamin ortholog Shibire (Shi) (Moline et al., 1999) with the myoblast-specific *1151*-Gal4 driver. Myoblasts were not obviously affected by these conditions, but stunted ASPs were observed in animals that expressed Rab5^DN^ or Shi^DN^ (Figure 6A–F). We also examined Notch signaling in animals with mutant myoblasts by monitoring Hnt and Su(H)lacZ expression. Both were reduced (Figure 6G–L). These results suggest that myoblast-produced Dl and the Dl-containing myoblast cytonemes activate Notch by the normal pathway of Notch signal transduction.

### Wg signaling in the wing disc represses Delta in the myoblasts

**Figure 7.**
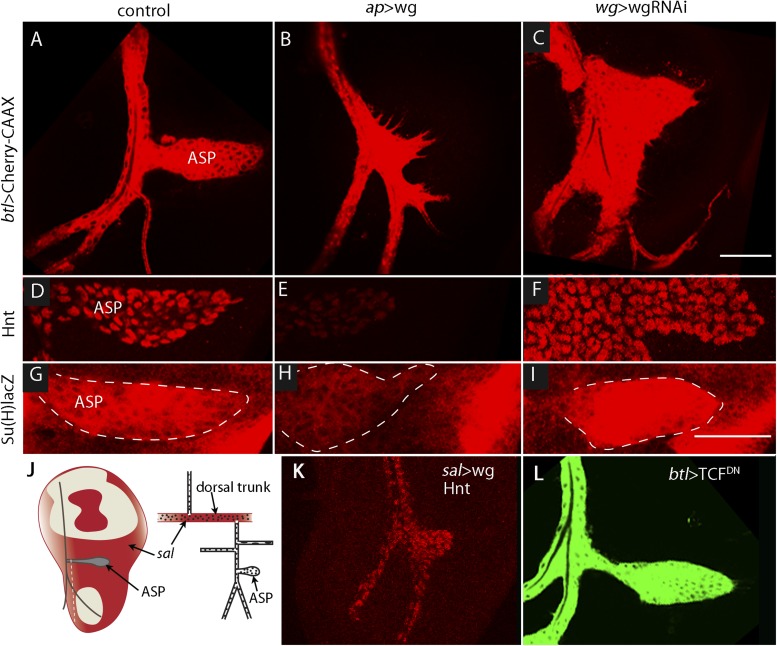
Wg expression in the wing disc affects ASP development and Notch signaling. Compared to controls (**A**, **D**, **G**), ectopic Wg expression (**B**, **E**, **H**) and wgRNAi expression (**C**, **F**, **I**) in the wing disc perturbed ASP development (**B** and **C**). Ectopic Wg expression reduced levels of Hnt (**E**) and Su(H)lacZ (**H**); wgRNAi increased Hnt and Su(H)lacZ (**F** and **I**). (**D**–**I**) Staining was with α-Hnt (**D**–**F**) and α-ß-galactosidase antibodies (**G**–**I**) and Alexa Fluor 555 secondary antibodies. (**J**) Drawings showing (left) areas of *sal* expression (red) in the wing disc (Grieder et al., 2009) and (right) Tr2 tracheal branches (Rao et al., 2015). Dashed white line indicates approximate location of sagittal sections shown in Figure 11E–H. (**K**) Over-expression of Wg in the *sal* domain reduced ASP development; (**L**) Expression of TCF^DN^ in trachea had no apparent effect on ASP development. (Genotypes: (**A**) *btl-LHG, lexO-Cherry:CAAX/+*; (**B**) *btl-LHG, lexO-Cherry:CAAX/ap-Gal4; UAS-wg/tub-Gal80*^*ts*^; (**C**) *wg-Gal4/UAS-wgRNAi; btl-LHG, lexO-Cherry:CAAX/UAS-wgRNAi*; (**D**) *wg-Gal4*/+; (**E**) *ap-Gal4*/+;*UAS-wg/tub-Gal80*^*ts*^*;* (**F**) *wg-Gal4/UAS-wgRNAi*;*UAS-wgRNAi*/+; (**G**) *Su(H)lacZ*/+; (**H**) *Su(H)lacZ*/+;*ap-Gal4/tub-Gal80*^*ts*^;*UAS-wg/+*; (**I**) *Su(H)lacZ/+;wgGal4/UAS-wgRNAi;UAS-wgRNAi/+*; (**K**) *sal-Gal4*/+;*UAS-wg/tub-Gal80*^*ts*^; (**L**) *btl-Gal4 UAS-CD8:GFP/UAS-TCF*^*DN*^). Scale bars: 50 μm. **DOI:** http://dx.doi.org/10.7554/eLife.06114.010

The sensitivity of Dl-dependent Notch signaling in the ASP implies that Dl expression in the myoblasts is regulated. To investigate how the abundance of myoblast Dl might be controlled, we carried out screens to identify genes whose mis-expression perturbed Notch signaling in the ASP. To search for genes whose expression in the wing disc affects the ASP, we expressed candidates in the dorsal disc cells (with *ap-*Gal4) while simultaneously marking the ASP (with *btl-*LHG *lexO-*mCherry-CAAX) and monitoring ASP growth and morphogenesis. Ectopic over-expression of Wg was found to severely reduce the ASP (Figure 7A,B,J,K) and to phenocopy Notch loss-of-function (Figure 3B). Conversely, reducing Wg below its normal levels in the disc by expressing *wg*-RNAi with a *wg*-Gal4 driver generated abnormally large and malformed growths (Figure 7C). These growths appeared similar to those produced by *btl >* Notch^CA^ flies (Figure 3C). Moreover, ASP expression of the Hnt and Su(H)lacZ Notch targets was decreased in wing discs with elevated levels of Wg (Figure 7D,E,G,H) and was increased in discs with decreased levels of Wg (Figure 7D,F,G,I). These results suggest that wing disc-produced Wg negatively regulates Notch signaling in the ASP.

**Figure 8.**
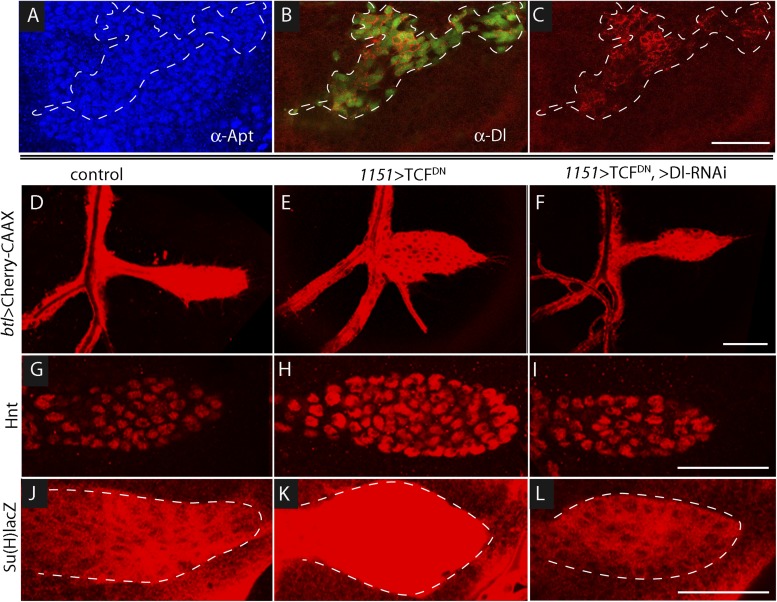
Wg signaling regulates the abundance of Delta in wing disc myoblasts. (**A**–**C**) A MARCM clone (Lee et al., 2000) of *dishevelled* mutant cells (outlined with dashed white line) expressed GFP (**B**) and up-regulated Dl (**C**). α-Apontic antibody staining identified myoblasts (**A**). Clone was induced by 1 hr heat shock 3–4 days after egg laying. (**D**–**F**) The ASP developed abnormally in the presence of TCF^DN^ in wing disc myoblasts (**E**) compared to control (**D**); the phenotype was suppressed by expression of DeltaRNAi in the myoblasts. (**G**–**L**) TCF^DN^ expression in the wing disc myoblasts increased Hnt (**H**) and Su(H)lacZ (**K**) relative to controls (**G** and **J**); DlRNAi reduced Hnt and Su(H)lacZ expression to control levels (**I** and **L**). (Genotypes: (**A**–**C**) *dsh*^*3*^ *FRT19A/hsFLP tub-Gal80*^*ts*^*,FRT19A;act-Gal4 UAS-GFP/+*; (**D**) *1151-Gal4/+;btl-LHG, lexO-Cherry:CAAX/+*, (**E**) *1151-Gal4/+;btl-LHG lexO-Cherry:CAAX/UAS-TCF*^*DN*^ and (**F**) *1151-Gal4/+;btl-LHG, lexO-Cherry:CAAX/UAS-TCF*^*DN*^*;UAS-DeltaRNAi/+*). Scale bars: 50 μm. **DOI:** http://dx.doi.org/10.7554/eLife.06114.011

To determine whether Wg produced by the wing disc signals directly or indirectly to the ASP, we expressed a dominant-negative form of the transcription factor Pangolin (TCF) in tracheal cells (with the *btl*-Gal4 driver). TCF^DN^ inhibits Wg signaling, but its expression in the ASP had no apparent effect (Figure 7L). This result suggests that Wg signaling is not activated in the ASP and that Wg does not signal directly to the ASP. To evaluate Wg signaling in the myoblasts, we generated clones of mutant myoblasts that were incapacitated for Wg signal transduction. *Dishevelled (dsh)* is an essential component of the Wg signal transduction pathway (Habas and Dawid, 2005), and although null *dsh* myoblasts did not activate Wg signal transduction, they grew to generate large clones (Figure 8A,B and Figure 8—figure supplement 1). However, their levels of Dl were elevated relative to *dsh*^*+*^ neighbors (Figure 8C). Ubiquitous expression of TCF^DN^ in myoblasts (with the *1151*-Gal4 driver) decreases the number of myoblasts (Sudarsan et al., 2001), but ASPs that developed in discs with these compromised myoblasts were large and deformed (Figure 8D,E). Increased Hnt and Su(H)lacZ expression in the abnormal ASPs (Figure 8G,H,J,K) indicates that Notch signal transduction was increased. These results suggest that Notch signaling in the ASP is sensitive to levels of Dl in myoblasts and that Dl expression in myoblasts is negatively regulated by Wg signaling. Genetic linkage between Wg signaling in myoblasts and Notch signaling in the ASP was demonstrated by simultaneously expressing dTCF^DN^ and Dl-RNAi in myoblasts (with the *1151-*Gal4 driver). ASPs in these double mutant discs were neither stunted nor misshapen (Figure 8D,F) and expression of Hnt and Su(H)lacZ was similar to controls (Figure 8G,I,J,L). These results imply that Wg signaling affects the ASP indirectly—that although Wg signaling may not be activated in the ASP, Wg signaling nevertheless plays an important role by down-regulating Dl expression in ASP-associated myoblasts that activate Notch signaling in the ASP. Wg-dependent down-regulation of Notch signaling has also been shown to be important for the myoblasts that generate the flight muscles of the adult fly (Gunage et al., 2014).

**Figure 8—figure supplement 1.**
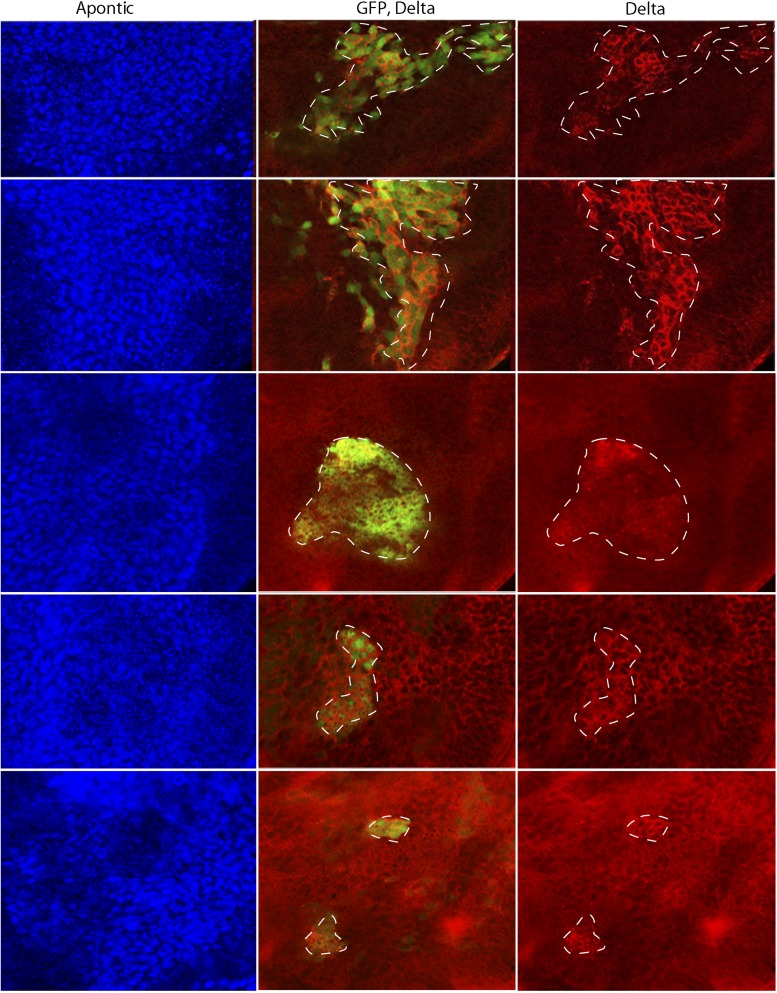
*disheveled* mutant clones up-regulate Delta expression. MARCM clones mutant for *dsh*^*3*^ in myoblasts (identified by expression of Apontic, left panels) that were induced 3–4 days after egg laying expressed GFP (middle panels) and increased Dl expression (right panels). Staining was with α-Apontic and α-Hnt antibodies. (Genotype: *dsh*^*3*^ *FRT19A/hsFLP tub-Gal80*^*ts*^*,FRT19A; act-Gal4 UAS-GFP/+*). **DOI:** http://dx.doi.org/10.7554/eLife.06114.012

### Wg in the notum signals to myoblasts in the early L3 stage

**Figure 9.**
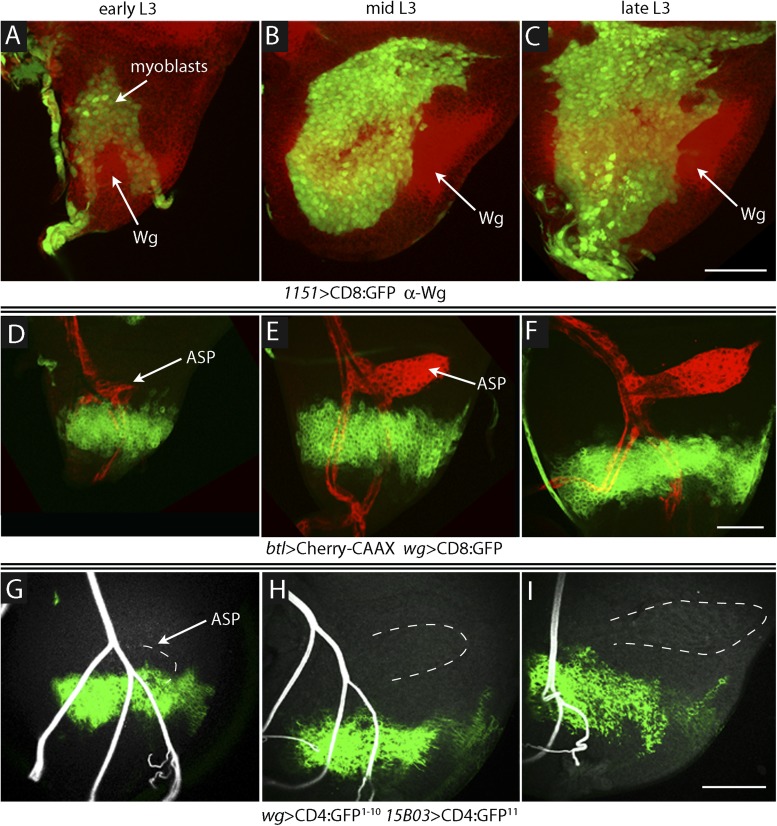
Proximity of Wg-expressing cells, myoblasts and ASP in the wing disc. (**A**–**C**) Images show the presumptive notum region of the wing disc during the third instar; the Wg domain (red) labeled by α-Wg antibody staining and myoblasts by GFP fluorescence (Genotype: *1151-Gal4 UAS-CD8:GFP*). (**D**–**F**) Images of the presumptive notum region of the wing disc show the ASP (red) and Wg-expressing disc cells (green) in early (**D**), mid (**E**) and late (**F**) L3 stage discs. (Genotype: *btl-LHG/wg-Gal4 UAS-CD8:GFP; lexO-Cherry:CAAX/+*). (**G**–**I**) Images show contacts (green fluorescence) between Wg-expressing disc cells and myoblasts generated by reconstituted GFP (GRASP). Auto-fluorescence of air-filled tracheal lumen was detected at 405 nm; perimeter of ASP is indicated by dashed white lines. (Genotype: *15B03-lexA/wg-Gal4;UAS-CD4-GFP*^*1–10*^ *lexO-CD4-GFP*^*11*^*/+*). Scale bars: 50 μm. **DOI:** http://dx.doi.org/10.7554/eLife.06114.013

**Figure 10.**
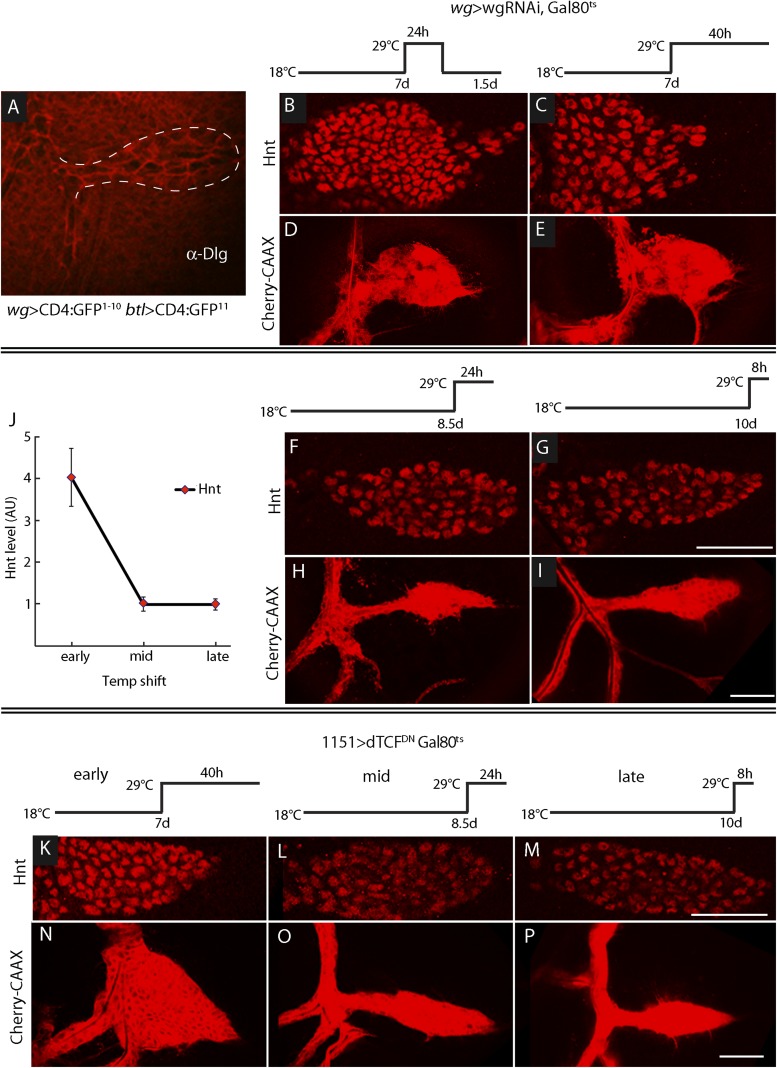
ASP development depends on Wg expression at the early third instar stage. (**A**) Image shows ASP (highlighted by α-Discs large antibody staining and outlined with dashed white line) from a GRASP experiment in which the fragments of GFP were expressed in the Wg domain of the disc and in the trachea. The absence of GFP fluorescence indicates that Wg-expressing disc cells did not directly contact the ASP. (Genotype: *wg-Gal4/lexO-CD4-GFP*^*11*^*;btl-LHG/UAS-CD4-GFP*^*1–10*^). (**B**–**I**) Expression of wgRNAi in the wing disc during the early L3 was induced as depicted in the line drawings above the images—either as a 24 hr temperature pulse at the non-permissive temperature for Gal80^ts^ (29°C) 7 days after egg laying (early L3, **B** and **D**), continuously at 29°C after 7 days (**C** and **E**), 8.5 days (mid L3, **F** and **H**) or after 10 days (late L3, **G** and **I**). wgRNAi perturbed ASP development (**D** and **E**) and increased Hnt expression (**B** and **C**) when expressed at early L3, but had no apparent effect on either Hnt expression (**F** and **G**) or ASP development (**H** and **I**) when expressed only at mid or late L3. (**F**, **G**, **H**, **I**) Staining with α-Hnt antibody was detected with Alexa Fluor 555 secondary antibody. (**J**) Quantitation of Hnt staining in (**B**, **C**, **F**, **G**) normalized to control (*wg > wgRNAi, Gal80*^*ts*^ at 18°C). N = 5 for each time point and control; error bars: standard deviation. (Genotypes: (**B**, **C**, **F**, **G**) *wg-Gal4/UAS-wgRNAi;UAS-wgRNAi/tub-Gal80*^*ts*^; (**D**, **E**, **H**, **I**) *btl-LHG lexO-Cherry:CAAX/wg-Gal4;UAS-wgRNAi/tub-Gal80*^*ts*^). (**K**–**P**) Expression of TCF^DN^ in myoblasts (with the *1151*-Gal4 driver) was induced as depicted in the line drawings above the images—either at the non-permissive temperature for Gal80^ts^ (29°C) 7 days after egg laying (early L3), 8.5 days (mid L3) or 10 days (late L3). ASP development was abnormal and Hnt staining increased with expression of TCF^DN^ at early L3, but had no apparent effect on either Hnt or ASP development when expressed only at mid or late L3. Scale bars: 50 μm. **DOI:** http://dx.doi.org/10.7554/eLife.06114.014

To investigate how Wg that is produced in the wing disc signals to the myoblasts and how Dl produced in myoblasts signals to the ASP, we first sought to understand where Wg-expressing disc cells are relative to myoblasts and the ASP. The number of myoblasts in early stage L3 discs is small, and we observed few of them directly overlayed Wg-expressing cells (Figure 9A). During L3, the myoblasts proliferate (Gunage et al., 2014) to cover most of the dorsal disc by late L3, and we observed that the domain of Wg-expressing cells in the dorsal disc also expanded (Figure 9B,C). However, the relative proximity of these Wg-expressing disc cells to the ASP decreased during the L3 period: although the most ventral Wg-expressing cells were within 5–10 μm of the ASP in early L3 discs, there were no Wg-expressing cells within 60–70 μm in late L3 discs (Figure 9D–F). In order to determine whether myoblasts contact Wg-expressing cells and where such contacts are relative to the ASP, we applied the GRASP technique. The fluorescence of the reconstituted GFP revealed extensive contacts between myoblasts and Wg-expressing disc cells throughout L3, but these contacting myoblasts were adjacent to the ASP only at the early L3 stage (Figure 9G–I). Wg-expressing disc cells did not contact the ASP or nearby tracheal cells at mid or late L3 (Figure 10A). These results suggest that Wg signaling from the disc to the ASP is indirect and that in the myoblasts, Wg signaling that is relevant to the ASP may be specific to the early L3 period.

To determine when the ASP requires Wg signaling in the myoblasts, we expressed *wg-*RNAi in the wing disc (with a *wg*-Gal4 driver) at various times during L3, and the ASP was analyzed for Hnt expression (to monitor Notch signaling) and morphology (Figure 10B–I). We used the Gal80^*ts*^ repressor to conditionally express the RNAi. At 18°C, the permissive temperature for Gal80^ts^, animals developed normally. However, expression of *wg*-RNAi at early L3, (7 days after egg laying at 18°C) affected both ASP morphology and Hnt levels. *wg*RNAi, expressed either for a pulse of 24 hr (between 7 and 8 days), or continuously (for 40 hr after 7 days), increased Hnt levels and resulted in ASPs that were blunted and enlarged (Figure 10B–E). In contrast, expression of *wg*-RNAi at mid (8.5 days after egg laying) and late (10 days after egg laying) L3 had no apparent effect on either Hnt levels or morphology (Figure 10F–J). Similar results were observed after ectopic expression of dTCF^DN^ in early, mid, and late L3 stages (Figure 10K–P). These results suggest that Wg signaling in myoblasts is required at the early L3 stage to control the level of Delta-dependent Notch signaling in the ASP and that the consequences of the early stage Wg signaling perdure to later stages.

**Figure 11.**
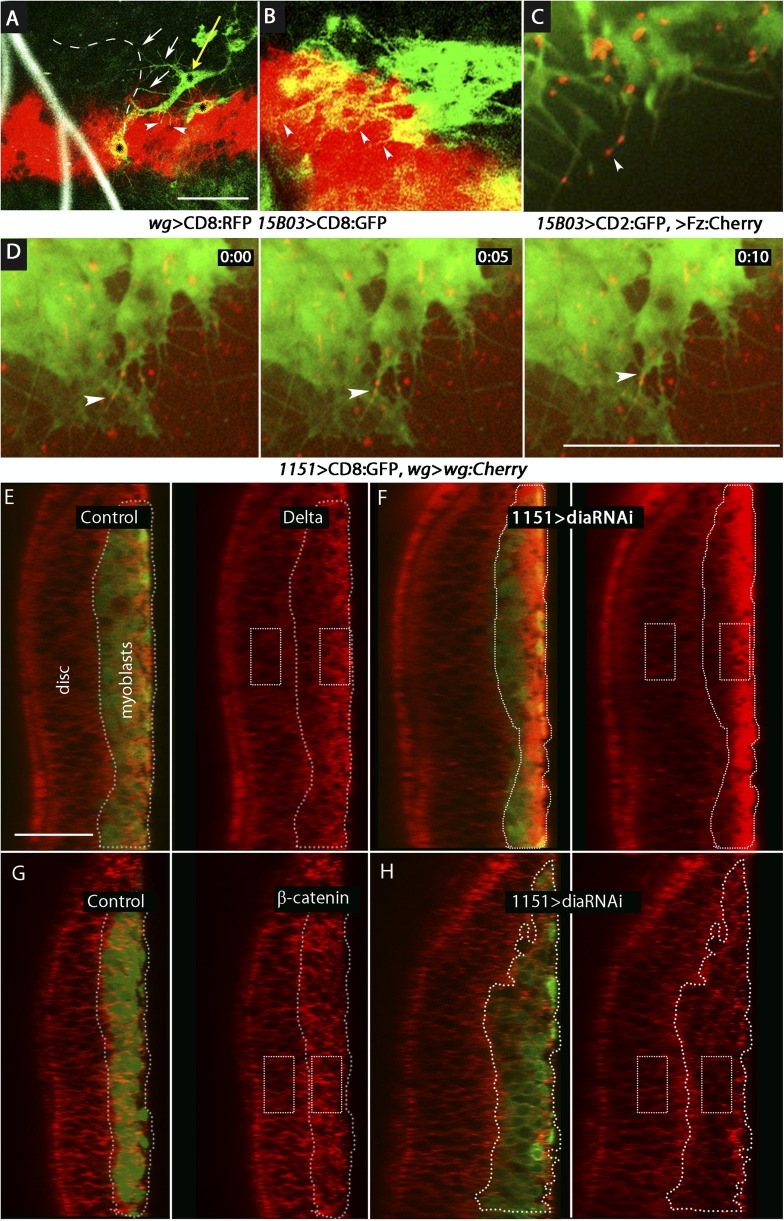
Delta and Wg localize to myoblast cytonemes. (**A** and **B**) Myoblast cytonemes (marked with CD8:GFP) at early L3. Images show Wg-expressing disc cells (red), the ASP (outlined by white dashed line in (**A**)), trachea (autofluorescence at 405 nm in (**A**)), and myoblasts (green, and indicated with black stars in (**A**)). Myoblast cytonemes extended toward the ASP (white arrows) and toward Wg-expressing cells (white arrowheads). Yellow arrow in (**A**) indicates a myoblast with both types of cytonemes. (**C**) Fz:Cherry puncta (white arrowhead points to one) visualized in myoblasts and myoblast cytonemes (marked with CD8:GFP). (**D**) Images obtained at 5 s intervals visualizing Wg:Cherry expressed in the Wg domain of the wing disc. Fluorescent puncta associated with disc cells (red background area), with myoblasts (green area) and with myoblast cytonemes (white arrowhead indicates a motile puncta). (**E**–**H**) Sagittal images showing wing disc and myoblasts (green; encircled by dotted lines) and stained with α-Delta (**E** and **F**) and α-β-catenin (**G** and **H**) antibodies (red) in the absence ((**E** and **G**) or presence (**F** and **H**) of *diaRNAi* expressed in myoblasts. Fluorescence intensity in the indicated boxes was measured using ImageJ and the levels in experimental samples relative to controls were calculated by comparing the ratio of myoblast to disc fluorescence. (Genotypes: (**A** and **B**) *UAS-CD8:RFP lexO-CD8:GFP/+;wg-Gal4, 15B03-LexA/+*; (**C**) *15B03-LexA lexO-Fz:Cherry/lexO-CD2:GFP*; (**D**) *1151-Gal4/+;wg:Cherry/+; UAS-CD8:GFP/+*; (**E** and **G**) *1151-Gal4/+;UAS-CD8:GFP/+*; (**F** and **H**) *1151-Gal4/+;UAS-CD8:GFP/UAS-diaRNAi*). Scale bars: 30 μm (**A**–**D**), 25 μm (**E**–**H**).) **DOI:** http://dx.doi.org/10.7554/eLife.06114.015

We designed two types of experiments to investigate how Wg moves from the disc cells to the myoblasts. We first marked the membranes of myoblasts with tethered GFP (using the *15B03*-*lexA* driver) in animals whose *wg*-expressing cells were marked with membrane-tethered RFP (using the *wg*-Gal4 driver). In early L3 discs, we observed that myoblasts extended cytonemes both toward *wg*-expressing cells and the ASP (Figure 11A,B). Myoblasts that had both types of cytonemes were detected (Figure 11A). We next investigated whether the myoblast cytonemes contain Fz and Wg. We analyzed preparations from larvae that expressed a Fz:Cherry protein fusion in myoblasts and whose cytonemes were marked with membrane-tethered GFP. Fz:Cherry was present in motile puncta, many of which localized to myoblast cytonemes and to cytoneme tips (Figure 11C and Video 2). We also analyzed preparations from larvae whose *wg*-expressing cells expressed a Wg:Cherry protein fusion and whose myoblasts expressed membrane-tethered GFP (driven by *1151-Gal4*). Many Wg:Cherry puncta were observed, some of which co-localized with and moved along GFP-marked myoblast cytonemes (Figure 11D and Video 3). To examine the role of the myoblast cytonemes, *dia*RNAi was expressed in the myoblasts, and Delta and β-catenin were monitored. In the presence of diaRNAi, levels of Delta increased (∼3×; Figure 11E,F) and levels of β-catenin decreased (∼0.6×; Figure 11G,H). Neuralized expression was also detected coincident with Delta (Figure 11–figure supplement 1). These results support the conclusion that myoblasts extend cytonemes that take up Wg directly from Wg-expressing disc cells.

**Figure 11—figure supplement 1.**
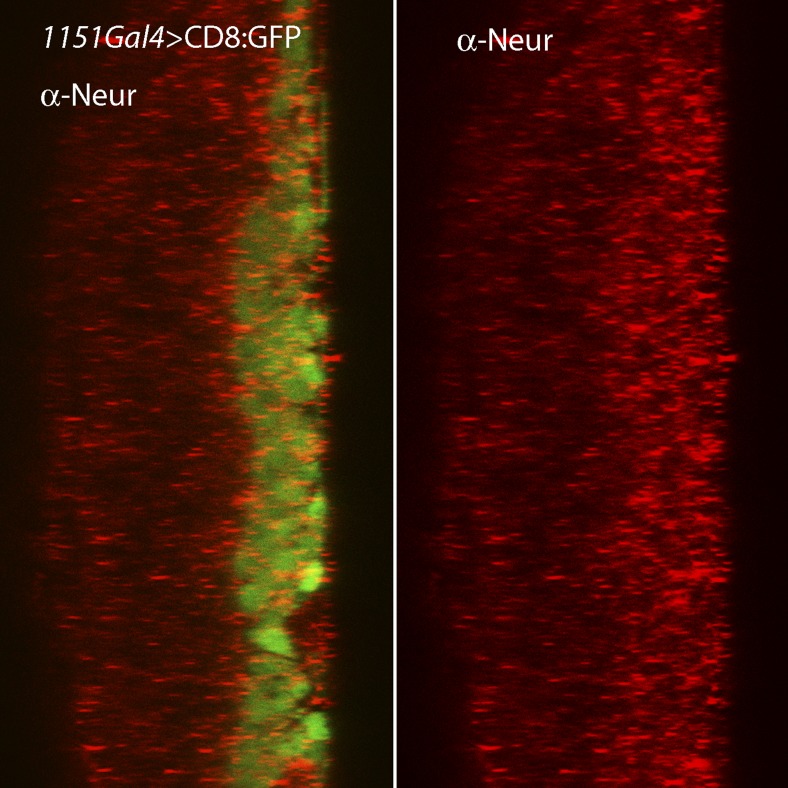
Expression of Neuralized in the wing disc and associated myoblasts. Staining with α-Neuralized antibody (red) detected elevated levels in the myoblasts relative to the disc epithelium. **DOI:** http://dx.doi.org/10.7554/eLife.06114.016

**Video 2.** Motile Frizzled receptors in the cytonemes. Fz:Cherry expressed in myoblasts was detected in fluorescent puncta that move along dynamic myoblast cytonemes (labeled with CD2:GFP). (Gentoype: *15B03-LexA lexO-Fz:Cherry/lexO-CD2:GFP*.) **DOI:** http://dx.doi.org/10.7554/eLife.06114.017

**Video 3.** Wg puncta is transported in myoblast cytonemes. Wg:Cherry expressed in the wg domain of the wing disc was detected in fluorescent puncta that move along myoblast cytonemes (labeled with CD8:GFP). (Genotype: *1151-Gal4/+;wg–wg:Cherry/+;UAS-CD8:GFP/+*.) **DOI:** http://dx.doi.org/10.7554/eLife.06114.018

## Discussion

These histological and genetic studies identified regulatory interactions and physical contacts between the wing disc epithelium, wing disc-associated myoblasts and the ASP that presage the functional relationships of the adult tissues they produce. Whereas some interactions between the disc and ASP are direct, such as the signaling by disc-generated Dpp and FGF that activate signal transduction in the ASP (Roy et al., 2014), our results now show that disc-dependent regulation of Notch signaling in the ASP is indirect. We found that the myoblasts are intermediaries that relay Wg signaling from the disc to control Notch signal transduction in the ASP.

There are precedents for intermediaries that link signaling pathways in different cells. In the wing disc, for example, Hh produced in the posterior compartment induces Dpp expression in a spatially limited group of anterior compartment cells that are situated just across the anterior/posterior compartment border. These cells function as the anterior/posterior organizer, dispersing Dpp protein that induces the expression of various target genes by cells across the disc. The Dpp-expressing cells may thus be considered to be intermediaries that relay Hh signaling. This relay system transforms a pattern of binary expression (Hh on in the posterior compartment, off in the anterior compartment) to the more complex nested patterns of expression of Dpp target genes.

The function of the myoblast relay system is not clear, and in contrast to the Hh-Dpp system, it may not be related to spatial patterns of gene expression. Key differences between the systems that may be relevant are timing and physical proximity. Hh induces Dpp expression at the anterior/posterior compartment border in the embryo and appears to do so continuously until the late L3. Moreover, the spatial relationship between the Hh and Dpp-expressing cells remains essentially unchanged as the disc grows and matures. In contrast, Wg signaling in the myoblasts is required only in early L3 (Figure 10) when the ASP and the Wg-expressing disc cells are relatively close (Figure 9). Myoblasts in this region facilitate Wg signaling by extending Fz-containing cytonemes that take up Wg. At later L3 stages, the patterns of Wg expression in the disc are quite different and the distance between Wg-expressing disc cells and the ASP is much greater (Figure 9). At these stages, the myoblasts that contact the ASP with Dl-containing cytonemes are juxtaposed to the ASP, far from the myoblasts that contact the Wg-expressing cells (Figures 2, 9, 10). The ASP has no apparent requirement for Wg signaling at these stages.

The late L3 ASP is a tubular structure that has a lumen and morphologically distinct stalk, medial, and tip regions that define a proximal/distal axis (Figure 1C). An orthogonal dorsal/ventral axis in the ASP is defined by cells that have contrasting levels of Dpp (Roy et al., 2014) and Notch (Figure 2) signal transduction in the ‘upper’ and ‘lower’ layers. These regional specializations materialize as the ASP grows during the mid and late L3 stages (Guha and Kornberg, 2005; Guha et al., 2009), but in early L3 when Wg signaling is needed, the ASP appears to be simply a small outgrowth with no discernable lumen or regional specializations. Our studies suggest that the myoblasts that activate Notch signaling in the lower layer cells of the late L3 ASP are defined by their prior exposure to Wg. We suggest that the myoblast relay system may accommodate the changing spatial relationship between the disc cells that express Wg and the ASP cells that require Notch signaling.

The framework for understanding the functions of homeotic genes in *Drosophila* has come from seminal studies that analyzed null mutants, temperature sensitive mutants, and somatic clones of mutant cells. The consistent finding that mutant cells respond similarly in the embryo and in the larval stages led to the general concept that homeotic genes are required continuously during development (Lawrence and Morata, 1994). Our observation here, that ASP requires Wg signaling only in early L3, is unusual in this context, but it is consistent with two prior reports. One described somatic clones in the wing disc that ectopically activated the Dpp pathway, and analyzed the expression of the Dpp target gene *optomotor-blind* (*omb*) (Lecuit et al., 1996). Differences between *omb* expression in the clones and in normal discs led to the proposal that some *omb*-expressing cells in late L3 discs are not active for Dpp signal transduction but express *omb* because of their exposure to Dpp at an earlier stage. The second study analyzed the expression of Wg target genes in wing discs that expressed a form of Wg that is fused to a transmembrane domain of an unrelated protein (Alexandre et al., 2014). It reported that Wg is normally expressed in a pattern of well-defined stripes in late L3 discs and more broadly in younger discs, and to reconcile the apparent discrepancy in late L3 discs between the restricted distribution of the membrane-tethered Wg (detected by immunofluorescence) and the broader expression domains of the Wg targets (*frizzled3* and *Distal-less*), it proposed that target genes that are induced in cells at early stages continue to be expressed by descendants of these cells that are not active for Wg signal transduction. Neither study directly examined the temporal dependence of signaling on target gene expression or characterized thresholds for the identified phenotypes.

Wg and its family of related Wnt proteins have inductive signaling activities in many contexts (Willert and Nusse, 2012), and the long-standing assumption has been that it is released into the extracellular aqueous environment in order to travel from producing to target cells. A requirement for cofactors that enhance solubility in vitro and promote long range signaling in vivo is consistent with this idea (Mulligan et al., 2012). However, the form of Wg that moves between pre- and post-synaptic cells at the *Drosophila* neuromuscular junction suggests a different mechanism. EM micrographs show that motoneurons secrete Wg in a vesicular form and that postsynaptic cells take up exosome-like vesicles that contain Wg (Korkut et al., 2009). We have previously reported that Dpp and FGF signaling in the ASP is mediated by cytonemes that synapse with signal-producing wing disc cells, and that several genes that are required at neuronal synapses also have essential roles at cytoneme synapses (Roy et al., 2014). We have also suggested that signaling at neuronal and cytoneme synapses is conceptually and structurally similar (Kornberg and Roy, 2014). Although cytoneme synapses that link Wg-producing wing disc cells and myoblasts have not been imaged with EM resolution, the most parsimonious model posits that cytoneme-dependent Wg signaling between disc cells and myoblasts is similar to Wg signaling at the neuromuscular junction and is also vesicle-mediated. The idea is that synaptic transfer and transit in exosome-like vesicles may be universal to Wg signaling. This reasoning has implications for signaling by membrane-tethered Wg.

The discovery that flies that depend on membrane-tethered Wg are viable and morphologically normal was interpreted as evidence that juxtacrine signaling by the tethered Wg is sufficient (Alexandre et al., 2014). An alternative possibility is that membrane-tethered Wg localizes to exosome-like vesicles that traffic along cytonemes and activates signal transduction while membrane-bound. Previous studies indicate that Dpp, FGF, EGF, Hh, and Notch may be cytoneme-mediated (Hsiung et al., 2005; Cohen et al., 2010; Callejo et al., 2011; Roy et al., 2011; Bischoff et al., 2013; Sanders et al., 2013; Chen and Kornberg, 2014; Roy et al., 2014). The results reported here add Wg to this list and provide evidence that Delta-Notch signaling is cytoneme-dependent. Exchange of signals at synapses may be a universal mechanism of paracrine signaling.

## Materials and methods

### *Drosophila* stocks

Flies were reared on standard cornmeal and agar medium at 22–25°C, unless otherwise stated. *btl-*Gal4, *btl-*LHG, *lexO-*CD2-GFP, and *UAS-*CD8:GFP were described (Roy et al., 2014). *lexO-CD4-GFP*^*11*^ and *UAS-CD4-GFP*^*1–10*^, from K Scott; *lexO*-mCherry-CAAX, from K Basler; *UAS-*CD4:IFP2.0-T2A-HO1 (Yu et al., 2014); *Wg-Cherry* knock-in, from JP Vincent; *UAS-*Notch^DN^, from N Perrimon (Micchelli and Perrimon, 2006), and *UAS-*Notch^CA^, from E Rulifson; *wg-*Gal4, from T Adachi-Yamada (Takemura and Adachi-Yamada, 2011); *wg-*RNAi (II and III), from the Vienna *Drosophila* RNAi Center stock center; Su(H)lacZ, from S Bray (Furriols and Bray, 2001); *1151*-Gal4, from K VijayRaghavan (Roy and VijayRaghavan, 1997); *sal-Gal4, UAS-GFP/CyO; Gal80*^*ts*^*/TM6b* (Makhijani et al., 2011).

### Immunohistochemistry and microscopy

Larvae were dissected in cold phosphate-buffered saline (PBS). Wing imaginal discs together with Tr2 trachea were fixed in 4% formaldehyde. After several washes, the discs were permeablized with TritonX-100 and blocked in 10% donkey serum. The following primary antibodies were used: α-Apontic (Eulenberg and Schuh, 1997), α-Hindsight, α-Wg, α-β-catenin, and α-β-galactosidase (Developmental Studies Hybridoma Bank, Iowa City, IA). Secondary antibodies (Jackson ImmunoResearch, West Grove, PA) were fluorescence-conjugated. Samples were mounted in Vectashield and imaged with a Leica TCS SPE confocal microscope.

### Live imaging techniques

Wing discs were dissected and placed in a drop of PBS underneath a coverslip using the ‘hanging drop’ method (Roy et al., 2014). Samples were imaged with a Leica TCS SPE or TCS SP2 confocal microscope with LAS-AF software. For time-lapse imaging, larvae were dissected in PBS and mounted in a slot formed between two strips of double sided tape, with the columnar layer facing the coverslip. Wing discs were incubated in Schneider's *Drosophila* medium containing fly extract, insulin, and penicillin-streptomycin. Images and videos were taken with a Nikon spinning-disc confocal microscope with 405 nm, 488 nm, 561 nm or 640 nm wavelength lasers. Videos were processed with NIS-Elements.

### EM microscopy

Larvae were dissected and fixed in 0.12 M Na-cacodylate buffer, pH 7.4 for 1 hr on ice. The wing discs were post-fixed with 1% OsO_4_ in 0.12 M Na-cacodylate buffer, rinsed with distilled water several times and incubated overnight in cold 2% uranyl acetate. Dehydration was with an ascending series of ethanol, infiltrated with Durcupan ACM resin and embedded in Durcupan resin. After polymerization, the resin blocks were sectioned by microtome. Images were captured with a FEI Tecnai12 TEM.

### Image quantification and statistical analysis

Cytonemes that extend to the ASP were scored. For each genotype, z-section stacks of confocal images from five ASPs were analyzed. The ratios in Figure 5H represent the number of cytonemes per unit length along the circumference of the ASP. To quantify Hnt staining (Figures 5I, 10J), the mean intensity of 555 nm fluorescence was measured in an area (containing approximately 20 cells) of the lower layer of the ASP. The value (with background fluorescence subtracted) was normalized with respect to Hnt levels in an area containing approximately 10 cells at the dorsoventral boundary of the wing disc. For each experiment, comparisons were made to control genotypes that were prepared and analyzed together with experimental genotypes in order to control for differences in staining and changes to laser intensity. Statistical significance was calculated by t-test.

### Molecular cloning

The Frizzled coding sequence was amplified from a cDNA clone (from P Adler) with primers: Forward: 5′-TCGAGAATTCCCAAAATGTGGCGTCAAATCC-3′ and Reverse: 5′-CGTCGAATTCAAGACGTACGCCTGCGCC-3′. Insert and lexO-Cherry vector were digested by EcoRI.
